# Scientific Medicine—A Medical and Surgical Review

**Published:** 1893-05

**Authors:** Thomas Wildes

**Affiliations:** Bluefields, Nicaraugua


### SCIENTIFIC MEDICINE—A MEDICAL AND SURGICAL REVIEW.

Thomas Wildes, M. D., Bluefields, Nicaragua.

The following is a critical review of allopathy—the miscalled "*scientific*," "*regular*," "*orthodox*" practice of medicine—by allopathic doctors, who are claimed to be judges in such matters, who are looked up to as authorities and teachers, and many of whom are the most eminent of the past and present.

The celebrated John Abernethy, founder of the distinguished School of St. Bartholomew's, and afterward principal surgeon of same; Professor of Anatomy and Surgery, Royal College of Surgeons; author of *Surgical Observations on the Constitutional Origin and Treatment of Local Diseases;* the first surgeon who had the courage to suggest and perform the daring operation of ligating the carotid and external iliac arteries; in his writings says:

"There has been a great increase of medical men of late years, but, upon my life, diseases have increased in proportion."

Francis Adams, A. M., M. D., LL. D., M. R. C. S., author of *Paulus Ægineta,* a busy practitioner, and the erudite translator of Hippocrates, says:

"We cannot think of the changes in professional opinions since the days of John Hunter without the most painful feelings of distrust in all modes of treatment."

Professor Claude Bernard, member of the French Institute, member Academy of Sciences, Professor of Physiology in the College of France, *vice* Magendie, deceased; discoverer of the glycogenic functions of the liver, said:

"Scientific medicine does not exist."

Professor Marie François Cavier Bichat, M. D., French anatomist, biologist, physiologist, physician, and author, wrote:

"Medical practice is said to be contradictory. I say more—it is not in any respect a profession worthy to be followed by sensible men."

Professor Archibald Billing, A. M., M. D., F. R. S., F. R. C. P. (London), a celebrated author and well-known London physician, lecturer at the London University of Medicine, member of the Senate and Examiner in Medicine at the same University, Vice-President Royal Medico-Chirurgical Society, Clinical Lecturer at the London Hospital, aired the following sentiment:

"Upon commencing the study of medicine I was appalled to find it a complete chaos."

Professor Hermann Boerhaave, M. D., a celebrated German doctor, and physician to Peter the Great of Russia, left behind him this record:

"If we weigh the good that has been done to mankind by a handful of true disciples of Æsculapius against the evil wrought to the human race by a great number of doctors since the origin of the art of medicine to our own time, we shall doubtless come to think that it would have been better had there never been any doctors in the world."

John Stileman Bostock, M. R. C. S., L. S. A., a learned English author, in his *History of Medicine*, says:

"And in the space of less than forty years we have gone through three revolutions of opinion with respect to our treatment of typhoid, a disease of very frequent occurrence and of the most decisive and urgent symptoms."

John Syer Bristowe, M. D., F. R. C. P., Physician and Lecturer on General Pathology at St. Thomas' Hospital, author, etc., jotted this down:

"Unfortunately a direct cure of disease by means of drugs is totally impossible in the great majority of cases."

Professor H. G. Cox, M. D., lectures that "Mercury is a sheet-anchor in fevers; but it is an anchor that moors your patient to the grave."

Professor E. S. Carr, M. D., of the New York University Medical School, has written:

"The effects of Mercury are not for a day, but for all time. It often lodges in the bones, and causes pain years after it is administered."

Sir Robert Christison, Bart., M. D., F. R. S., LL. D., Professor of Materia Medica, University of Edinburgh, President of the Royal College of Physicians, Edinburgh, Physician in Ordinary to the Queen, etc., etc., in his inaugural address to the graduates, said:

"Of all the medical sciences, therapeutics is the most unsettled and unsatisfactory in its present state, and the least advanced in its progress."

Professor William Cullen, M. D. (Glasgow), famous teacher, lecturer, and author; author of the world-renown *Treatise on Materia Medica*, published the following:

"Our *Materia Medicas* are filled with innumerable false deductions, which are nevertheless said to be derived from experience."

John Mason Good, M. D., F. R. S., author of *The Study of Medicine*, in that work says:

"The science of medicine is a barbarous jargon, and the effects of our medicines on the human system are in the highest degree uncertain; except, indeed, that they have destroyed more lives than war, pestilence, and famine combined."

Professor C. A. Gilman, M. D., says:

"A mild mercurial course and mildly cutting a man's throat are synonomous terms."

Dr. Krueger Hansen, a great medical authority, writes:

"Medicine, as now practiced, is a pestilence to mankind; it has carried off a greater number of victims than all that murderous wars have ever done."

Charles Kidd, M. D., M. R. C. S., author of various treatises on *The Use of Anæsthetics*, on *Gun-shot Wounds*, etc., etc., wrote:

"It is a sad and humiliating confession that at present our chiefest hopes of medical reform exist in the *outer* educated public."

Dietrich George Kieser, M. D., a great German doctor and a highly esteemed authority in medicine, says:

"In many cases the old saying holds good that the remedy is worse than the disease, and the doctor does more mischief than the malady."

Professor François Magendie, M. D. (Paris), member of the French Institute of Medicine, celebrated physiologist and teacher, wrote and lectured as follows:

"Let us no longer wonder at the lamentable want of success which marks our practice when there is scarcely a sound physiological principle amongst us. I hesitate not to declare, no matter how sorely I should wound our vanity, that so gross is our ignorance of the real nature of the physiological disorder called disease that it would, perhaps, be better to do nothing and resign the complaint into the hands of nature than to act as we are frequently compelled to do, without knowing the why and wherefore of our conduct, at the obvious risk of hastening the end of the patient." And in addressing his medical class, he says: "Gentlemen, medicine is a great humbug. I know it is called science. Science, indeed! It is nothing like science. Doctors are merely empirics when they are not charlatans. We are as ignorant as men can be. Who knows anything in the world about medicine? Gentlemen, you have done me the honor to come here to attend my lectures, and I must tell you frankly now, in the beginning, that I know nothing in the world about medicine and I don't know anybody who does know anything about it. * * * I repeat it, nobody knows anything about medicine. * * * We are collecting facts in the right spirit, and I daresay, in a century or so, the accumulation of facts may enable our successors to form a medical science. Who can tell me how to cure the headache, or the gout, or diseases of the heart? Nobody. Oh! you tell me doctors cure people. I grant you people are cured, but how are they cured? Gentlemen, nature does a great deal; imagination a great deal; doctors—devilish little, when they don't do any harm! Let me tell you, gentlemen, what I did when I was physician at the Hotel Dieu. Some three or four thousand patients passed through my hands every year. I divided the patients into two classes; with one I followed the dispensary and gave the usual medicines, without having the least idea why or wherefore; to the others I gave bread pills and colored water, without, of course, letting them know anything about it; and occasionally, gentlemen, I would create a third division to whom I gave nothing whatever. These last would fret a great deal; they would feel that they were neglected; sick people always feel that they are neglected, unless they are well drugged, *les imbeciles*, and they would irritate themselves until they got really sick, but Nature invariably came to the rescue, and all the third class got well. There was but little mortality amongst those who received the bread pills and colored water, but the MORTALITY WAS GREATEST among those who were carefully DRUGGED according to the DISPENSARY."

Wm. O. Markham, M. D., F. R. C. P., F. K. Q. C. P., author of a prize essay on the *Surgical Practice of Paris*, and of a *Treatise of Diseases of the Heart*, in his address on *Medicine*, says: "It may be cruel, and humiliating to the pride of medicine, to acknowledge that from the days of Hippocrates to our own it has put faith in and practiced the most grievous errors."

Professor Martin Payne, M. D., says: "Drugs do but cure one disease by producing another."

The celebrated Dr. Ramage says: "It cannot be denied that the present system of medicine is a burning shame to its professors, if, indeed, a series of vague and uncertain incongruities deserves to be called by that name. How rarely do our medicines do good! How often do they make our patients really worse! I fearlessly assert that, in most cases, the sufferer would be safer without a physician than with one. I have seen enough of the malpractice of my own professional brethren to warrant the strong language I employ."

Dr. Reid: "More infantile subjects are perhaps destroyed by the mortar and pestle than in the ancient Bethlehem fell victims in one day to the Herodian massacre."

Professor Reil: "Our knowledge of the effects of medicine is empirical."

Dr. Adam Smith: "The great success of quacks in England has been altogether owing to the real quackery of the allopathic physicians."

Thomas Smith, F. R. C. S., Surgeon to St. Bartholomew's Hospital, late Honorary Secretary Royal Medico-Chirurgical Society, wrote: "Medicine, as a whole, as it comes to us, is not a science properly so called, and has none of the exact laws of true science, nor are its doctrines capable of demonstration."

The venerable Professor Alexander H. Stephens, M. D., of the New York College of Physicians and Surgeons, in a lecture to his medical class, says: "The older physicians grow, the more skeptical they become of the virtues of medicine, and the more they are disposed to trust to the powers of nature." Again, "notwithstanding all our boasted improvements, patients suffer as much as they did forty years ago."

Hahnemann has sometimes been blamed for the severity of his criticisms upon the absence of therapeutics in the allopathic school, but he never said anything half so severe as many of the criticisms given above from the pens of professional allopaths; nor have the most ardent champions of Homœopathy ever spoken of allopathy in such opprobrious terms as we here see its leading men speaking of it. Hence when allopaths tell us that they are bitterly opposed to Homœopathy on account of its unscientific character, its impotence to cure disease, its absurdities and its opposition to all the experience of the two thousand five hundred years during which their own miscalled *Scientific* system has existed, they simply hurl a boomerang which recoils upon their own illogical skulls, and leaves Homœopathy progressing unharmed!

Each and every one of the above quoted men is now, or was, an eminent allopath; and if such distinguished gentlemen can thus publicly proclaim their ignorance, how about the average allopath who only knows enough of medicine to enable him to abuse Homœopathy, the science of therapeutics—a science which he has not the brains to comprehend!

Inasmuch as this was written in the Island of Jamaica, where yellow fever is endemic all the year round, and sometimes epidemic, and where all who are attacked with it and are treated allopathically are liable to die, and nearly all so treated do die, I add the following quotation from the *Daily News Letter* of September 3d, 1890, for the sake of contrast:

MEDICAL—YELLOW FEVER IN FLORIDA, U. S. A.

(September 8th, 1892. Written September 6th, 1890).

Henry R. Stout, M. D., Jacksonville, Florida, U. S. A., in his official report to the American Institute, concerning the yellow fever epidemic which began in August 8th, 1888, says that "the allopaths largely adhered to their germicidal treatment with *bichloride of mercury*, often prescribing it in the most reckless manner, causing dysentery and exhaustion with frequent disastrous results to the patient. One physician reported a mortality of twenty-eight per cent." "Under homœopathic treatment the disease is no more to be feared than an ordinary remittent fever. That such is the case is capable of demonstration from the books of the Board of Health of Jacksonville, Florida. There were reported 4,696 cases, with 430 deaths, a mortality of 9.2 per cent. Of this total number of cases 2,173 were white, with 331 deaths, a mortality of 15.2 per cent. The mortality among the negroes was 4 per cent. At Sand Hill hospital 216 cases were treated with 34 deaths, a mortality of 15.7 per cent. There were reported 501 cases treated homœopathically by Drs. P. E. Johnson, C. W. Johnson, and Henry R. Stout, with 13 deaths, a mortality of 2.6 per cent.—being less than one-third of the total general mortality. Included in these thirteen deaths is one case that had been previously overdosed by *bichloride of mercury*, and one that had been abandoned in a hopeless condition by an allopath. Each case was reported to the Board of Health by the visiting physician within a few hours after his first visit to the patient, giving the patient's name, age, residence, etc. If death followed, then the death certificate also gave name, age, residence, etc."

The foregoing cases of extreme fatuity and "unconscious bias" were in my original manuscript, but were of necessity omitted from the Jamaica newspaper article for want of space. The portion then printed has been copied into medical journals of the United States, but is incomplete without this, and, as a whole, makes a powerful arraignment of the *unscientific* old school of medicine, which steals our thunder, and then abuses us for making thunder, and maligns us for having any originality of thought.

If you would fully appreciate the ignorance and brutality of the average allopath you should go to Jamaica and encounter those half-educated, or so-called "licentiates," *English* "doctors" who have no diploma but have plenty of supercilious insolence.

The allopaths show us no mercy. We should give them no quarter, and the sooner our President issues a proclamation forbidding English and German doctors to practice in America the sooner will their law-makers come to their senses, and recognize American diplomas, as well as use American text-books in their so-called colleges, as now.

I desire to acknowledge my indebtedness to *Homœopathic League Tract, No.* 9, and to a publication entitled *Scientific Opinions on Drug Medication and Allopathy,* kindly sent me by Dr. James Gordon Bennett, of Halifax, Nova Scotia. I have merely culled and compiled, with the addition of a little fresh material, and made the document unanswerable and incapable of any disputation, by spending time and labor in hunting up the numerous titles of these fatuituous gentlemen.

